# An Epidemic of Virulent Diphtheria

**Published:** 1929

**Authors:** Thomas Aubrey, R. B. Britton

**Affiliations:** Medical Officer of Health to Mangotsfield Urban and Warmley Rural District Councils


### AN EPIDEMIC OF VIRULENT DIPHTHERIA.

BY

THOMAS AUBREY, M.B.,
*Medical Officer of Health to Mangotsfield Urban and Warmley Rural District Councils.*

AND

R. B. BRITTON, M.B., B.S.

---

THE comparatively small but seriously lethal epidemic of virulent or "malignant" diphtheria described in this article exhibited certain unusual clinical and bacteriological characteristics which were thought to be of sufficient general interest to warrant recording. The cases comprised a series of 40 in number from the middle of January, 1929, to May 14th, 1929. There were 22 males and 18 females. The ages ranged from twenty-five to two years, but 52 per cent. were under ten years, and, curiously, 25 per cent. were in their eighth year. The cases arose in a large and scattered area, and no particular school or carrier or milk supply could be proved responsible for the outbreak.

*Symptoms.* — The membrane was unusually extensive, involving tonsils, fauces, uvula and soft palate, and in some cases the naso-pharynx. It always presented a peculiar, slimy and semi-transparent appearance, very much resembling the scales of a fish. Microscopically, swabs were invariably reported negative for the Klebs - Lœffler bacillus, but were swarming with streptococci. An even more curious fact, which, however, is explained later by the result of post-mortem examinations conducted in two cases, was that direct microscopical and cultural examinations

M

of large pieces of detached membrane were also returned negative for the Klebs - Lœffler bacillus and positive for streptococci.

A constant feature was marked cervical adenitis and peri-adenitis, so that the neck presented a "bull-neck" appearance. There was always an extremely fœtid odour from the throat, and nasal discharge was always profuse and in most cases hemorrhagic.

Toxæmia was severe, as evidenced by the dullness and apathy of the patient and the appearance of definite purpuric spots on the skin and the occurrence of ecchymoses around the site of the serum injections.

Two fatal cases of laryngeal diphtheria occurred in our own private practices, and we have heard of another in that of a neighbouring practitioner. All these were fatal in one to three days.

*Treatment.*—On the assumption that these cases were regarded as a very virulent form of diphtheria dominated by a secondary streptococcal infection, the combined use of anti-diphtheritic and anti-streptococcal sera was instituted. As a routine 40,000 units of diphtheria antitoxin and 3,000 units of anti-scarlet-fever globulins (B. W. & Co.) were given intramuscularly on admittance. The diphtheria antitoxin was repeated in twelve hours, if there was any sign of extension of the membrane or increase in the toxæmia. The anti-scarlet-fever serum was repeated every twenty-four hours for three successive days. In some cases as much as 104,000 units of antitoxin was administered. The other items consisted in keeping the patient absolutely flat in bed and avoiding the slightest exertion.

*Results.*—Ten patients died and thirty recovered, the mortality, therefore, being 25 per cent. The deaths were either early from toxæmia, or late from myocarditis as evidenced by dilatation of the heart, extra-systoles, "gallop-rhythm" and cardiac vomiting.

Palatal paralysis occurred in 17 cases (42 per cent.), ciliary paralysis in 5 cases (14 per cent.), and otitis in 7 cases (17 per cent.). Broncho-pneumonia occurred in one case, that of an infant of two years, and ended fatally. No actual paralysis of the lower extremities was seen, but two patients lost their knee-jerks. Oculomotor paralysis did not occur in any case of the series.

*Post-mortem findings.*—Professor Walker Hall, of the Department of Pathology in the University of Bristol, performed an autopsy on one of the fatal cases, that of a male aged eight years. He found superficial sloughs and hemorrhages in the tonsils, but the larynx and trachea were free from any typical croupous exudate. The lungs did not show any areas of consolidation, and the heart, including valves and myocardium, was normal. The alimentary tract, liver and kidneys did not present any feature of note.

Cultures from the upper surface of the tonsil yielded streptococci, but Klebs-Lœffler bacilli did not grow out. Cultures from the trachea yielded streptococci and atypical diphtheria bacilli. Blood cultures taken from the heart blood were negative in every instance.

Cultures from the upper surface of the tonsil were injected into one guinea-pig, and cultures from the trachea into another. Both animals died on the fourth day. Both showed hemorrhages into the pleuræ and hemorrhagic suprarenals. A third guinea-pig was injected with the material grown from the deeper part of the tonsil. This animal lived for fourteen days, and did not show any evidence of diphtheritic intoxication.

Guinea-pig A was injected with an emulsion from the trachea and tonsils + 800 units of diphtheria antitoxin, and was killed on the seventh day. It showed an absence of any local lesion, but a few hemorrhages into the suprarenals.

Guinea-pig B was injected with an emulsion from the trachea and tonsils + 0·5 cc. of scarlatinal antiserum. This was killed on the seventh day, and showed entire absence of any local or general lesion.

Guinea-pig C was injected with an emulsion from the trachea and tonsils + 800 units of diphtheria antitoxin + 0·5 cc. of scarlatinal antiserum. This was killed on the seventh day, and showed no sign of local or general lesion.

These findings show that death was due to a mixed infection of streptococci and diphtheria bacilli. Diphtheria antitoxin failed to give complete protection, but this was obtained when anti-scarlatinal serum was added.

